# Psychological interventions for adult earthquake-related post-traumatic stress disorder: systematic review and meta-analysis

**DOI:** 10.1192/bjo.2025.784

**Published:** 2025-06-05

**Authors:** Cate F. Woods, Ben Beaglehole, Christopher Frampton, Virginia V. W. McIntosh, Caroline Bell

**Affiliations:** School of Psychology, Speech and Hearing, University of Canterbury, Christchurch,New Zealand; Department of Psychological Medicine, University of Otago, Christchurch, New Zealand

**Keywords:** Earthquake, post-traumatic stress disorder, psychological intervention

## Abstract

**Background:**

A minority of earthquake-exposed individuals develop post-traumatic stress disorder (PTSD), often alongside comorbid depression and anxiety symptoms. No systematic review has examined psychological interventions for adults with substantial earthquake-related PTSD symptoms.

**Aims:**

To synthesise studies evaluating psychological interventions for adult earthquake-related PTSD and conduct meta-analyses estimating overall effect sizes.

**Method:**

The review was pre-registered with PROSPERO (CRD42023441020). PsycINFO, MEDLINE, EMBASE, CINAHL and Scopus were searched for studies (last search conducted July 2024). Randomised controlled trials (RCTs), non-randomised and non-controlled studies evaluating psychological interventions for adults with substantial earthquake-related PTSD symptoms were eligible. Outcomes were PTSD, depression and anxiety symptoms. Narrative syntheses and meta-analyses summarised study findings. The Mixed Methods Appraisal Tool guided quality assessments.

**Results:**

Sixteen studies were identified (eight RCTs, four non-randomised and four non-controlled studies), representing 1315 participants receiving psychological intervention. Interventions included cognitive behavioural therapy (CBT), specific CBT variants, eye movement desensitisation and reprocessing, interpersonal psychotherapy and an internet-based intervention focusing on social cognitive theory. Studies generally reported statistically and clinically significant improvements associated with psychological interventions. Among studies included in meta-analyses, overall effect size was 2.11 (95% CI = 0.92, 3.31) for PTSD symptoms and 1.01 (95% CI = 0.50, 1.52) for depression symptoms.

**Conclusions:**

Psychological interventions are associated with good outcomes among adults with earthquake-related PTSD. The most evidence currently exists for CBT-based interventions, which are recommended as first-line treatments. Efficient intervention options, including single-session and group-based treatments, also show promise and are recommended for addressing widespread treatment need.

Major earthquakes are collectively experienced events that can result in deaths, injuries and widespread damage.^1^ Most earthquake-exposed individuals do not develop ongoing mental health issues. However, a substantial minority do, including post-traumatic stress disorder (PTSD),^2^ depression,^3^ anxiety disorders^4^ and subthreshold symptoms of these disorders.^5^ PTSD is characterised by symptoms of intrusion, avoidance and hyperarousal, alongside disturbances in mood and cognition.^6^ Incidence estimates of post-earthquake PTSD (assessed using clinician-administered interviews) have ranged from 4.4 to 42.0%.^2^ Clinical guidelines recommend psychological interventions as first-line treatments for PTSD, including cognitive behavioural therapy (CBT), specific CBT variants (cognitive therapy, cognitive processing therapy and prolonged exposure therapy) and eye movement desensitisation and reprocessing (EMDR).^7,8^

Several syntheses have evaluated psychological interventions for PTSD following natural disasters, and have generally found these interventions to be effective for this population.^9–11^ However, earthquakes are distinct from other natural disasters, such as major storms, floods and wildfires. Earthquakes often occur without warning and are associated with ongoing trauma exposure in the form of recurring aftershocks, which may persist for months or years. Consistent with this, some evidence suggests that earthquake trauma is associated with distinct features, including strong conditioned fear responses and helplessness, both of which may develop in response to ongoing, uncontrollable exposure to aftershocks.^12^ The unique characteristics of earthquakes, and potentially of earthquake-related PTSD, could influence the efficacy of psychological interventions. For instance, implementing exposure-based treatments may be challenging when there is ongoing threat posed by recurring aftershocks.^13^

Furthermore, natural hazards in and of themselves do not result in disasters (the term ‘natural disaster’, however, remains prevalent in the disaster mental health literature). Rather, disasters occur when these hazards interact with conditions of the built environment (e.g. inadequate building regulations) and social circumstances (e.g. socioeconomic vulnerability).^14^ While the frequency of earthquakes has remained stable,^15^ population growth in areas with high risk of seismic activity, combined with increased urbanisation in lower- and middle-income countries, is likely to heighten their impact,^1^ making the treatment of earthquake-related PTSD an important public health priority.

A recently published systematic review and meta-analysis^16^ synthesised research evaluating clinician-administered psychotherapies for earthquake-related PTSD. However, this review had several limitations. It was not pre-registered, did not include several potentially relevant studies,^17–22^ excluded studies that assessed PTSD categorically, included studies in which participants were not required to have PTSD symptoms, did not conduct formal quality assessments and did not examine the influence of psychological interventions on comorbid symptoms. Additionally, the review’s findings were not specific to adult populations, with approximately half of the included studies focused on children and/or adolescents.

The current systematic review addresses these gaps and provides an up-to-date analysis by synthesising RCTs, non-randomised and non-controlled studies evaluating psychological interventions for adults formally diagnosed with earthquake-related PTSD, or with substantial earthquake-related PTSD symptoms (e.g. surpassing a clinical cut-off on a self-report measure of PTSD severity). In addition to PTSD severity, we also examined their influence on comorbid depression and anxiety symptoms and conducted quality assessments to assess the strength of the available evidence.

## Method

The review was pre-registered with PROSPERO (CRD42023441020) and can be accessed at https://www.crd.york.ac.uk/prospero/display_record.php?RecordID=441020. The Preferred Reporting Items for Systematic Reviews and Meta-Analyses (PRISMA) checklist^23^ guided the review and can be found in Supplementary Table 1. Ethical approval and informed consent were not sought because the study was a systematic review of previously published studies.

### Eligibility criteria

The review included RCTs and non-randomised and non-controlled studies evaluating psychological interventions for earthquake-related PTSD. Non-randomised and non-controlled studies were included because a relatively small number of RCTs were anticipated, and excluding these studies may have resulted in a loss of valuable information. Case studies and case series were excluded. Studies were eligible if they included participants aged 16 years or older with either a formal PTSD diagnosis or substantial PTSD symptoms (e.g. surpassing a clinical threshold on a measure of PTSD severity) related to earthquake exposure. The initial review protocol specified that studies were eligible if they focused on participants aged 18+ years. Following preliminary database searches, however, several studies of adult participants were identified that used a slightly lower age threshold (i.e. 16 years). It was decided to include these studies because they were of direct relevance to the review, and contained samples consisting largely of participants aged 18+ years. Studies utilising continuous outcome measures were required to report pre- and post-treatment means and standard deviations for calculation of standardised mean differences (SMDs). Studies focusing on child and/or adolescent participants (aged 15 years or younger), or on participants without PTSD or PTSD symptoms, were excluded. The review focused only on psychological interventions for PTSD; studies of other interventions (e.g. pharmacological, psychosocial or alternative interventions) and interventions targeting other disorders (e.g. depression) were excluded.

### Search strategy

The search strategy was developed in consultation with a specialist research librarian. Five databases (PsycINFO, MEDLINE, EMBASE, CINAHL and Scopus) were searched for relevant studies. PsycINFO and CINAHL were accessed using the EBSCO interface; Embase and MEDLINE were accessed using the Ovid interface. Subject headings and keywords relating to psychological interventions, PTSD and earthquakes were combined using the Boolean classifier AND. No filters or date limits were used. The full search strategy can be found in Supplementary Table 2. Initial searches were run on 14 September 2023, and rerun on 20 July 2024 prior to data synthesis. The reference lists of prior reviews and eligible studies were manually searched to identify relevant studies not captured in the database search.

Two reviewers (C.F.W. and C.B.) independently screened articles identified through the search process using Covidence, a web-based platform for systematic review management.^24^ Reviewers first screened titles and abstracts (percentage agreement, 86%; Cohen’s kappa = 0.47), then progressed to full-text screening (percentage agreement, 68%; Cohen’s kappa = 0.42). Disagreements at both stages were resolved through discussion, or with a third reviewer (VVWM) if consensus could not be reached through discussion. If additional information was required to determine study eligibility, corresponding authors were contacted by email.

### Data extraction

Data were extracted from eligible studies between 14 and 16 January 2025. The primary outcome of interest was pre- to post-treatment change in PTSD symptoms, assessed using either a continuous measure of symptom severity or a diagnostic interview assessing the presence of a PTSD diagnosis. Secondary outcomes were change in depression and anxiety symptoms. For each study, the following information was extracted: study design; number and timing of assessments; participant characteristics; intervention characteristics; and pre- and post-treatment means and standard deviations for measures of PTSD, depression and anxiety symptoms (or rates of diagnoses), alongside results of relevant statistical tests. For RCTs and non-randomised studies with multiple post-treatment assessments, results were extracted for all assessments with a corresponding between-group comparison. For non-controlled studies with multiple post-treatment assessments, results for all assessments were extracted. Extraction of key information, including that used to calculate effect sizes (see below) and results of relevant statistical analyses, was completed independently by two reviewers (C.F.W. and C.B.), with any discrepancies resolved through discussion.

#### Calculation of effect sizes

Standardised mean differences were calculated to assess the magnitude of treatment effects and facilitate between-study comparisons. For non-controlled studies, Cohen’s *d* was calculated by dividing the mean change (between pre- and post-intervention scores) by the standard deviation of the change, and reflects the magnitude of within-group pre- to post-treatment change. For RCTs and non-randomised studies, SMDs were calculated using the mean change and estimate of the standard deviation of the change for the intervention and control/comparison group, and reflects the magnitude of between-group difference in pre- to post-treatment change. Because only one study reported the standard deviation associated with within-group mean change scores (and none reported the correlation between pre- and post-treatment scores), the standard deviation of the change was derived from reported within-group effect sizes, from *t*-values testing the change within groups, or from confidence intervals associated with mean change, where possible. For studies that did not report information from which the standard deviation of the change could be derived, the larger of the pre- or post-treatment standard deviations was used.

#### Quality assessment

The Mixed Methods Appraisal Tool (MMAT)^25^ was used to assess the methodological quality of the included studies. The MMAT was chosen because it contains quality assessment criteria for multiple study designs, allowing for the use of a single tool across the RCTs and non-randomised and non-controlled studies. For RCTs, the MMAT assesses whether randomisation was performed appropriately, whether groups were comparable at baseline, degree of complete outcome data, blinding of outcome assessors and adherence to the assigned intervention. For non-randomised and non-controlled studies, the tool assesses sample representativeness, appropriateness of outcome measurements, degree of complete outcome data, whether confounders were adequately accounted for and whether the intervention was administered as intended. Each domain is rated as ‘Yes’ or ‘No’ or, alternatively, as ‘Can’t tell’ if insufficient information was reported for a judgement to be made. The MMAT is not scored. Two reviewers (C.F.W. and C.B.) independently completed quality assessments, resolving disagreements through discussion.

### Synthesis

#### Narrative syntheses

Narrative syntheses were used to summarise the findings of the included studies, in the following key categories: (a) study and sample characteristics, including study design, type of control/comparison condition, country in which study was conducted and sample age and gender distributions; (b) intervention type and characteristics, including descriptions of the evaluated interventions, intervention length and format; and (c) intervention outcome, grouped by study design. Results of quality assessments were tabulated to provide a visual summary of the quality of the included studies.

#### Meta-analyses

Meta-analyses were conducted on a subset of the included studies that shared similar methodological characteristics. Studies that compared a psychological intervention with a control group receiving no intervention, and those that reported pre- and post-treatment means and standard deviations (or other information allowing for the standard deviation of the change to be derived) for a continuous measure of PTSD or depression symptom severity, were included in meta-analyses. Too few studies included a measure of anxiety symptoms to conduct meta-analyses for this outcome. Random effects meta-analyses were conducted using SPSS v.29.0 to calculate pooled SMDs and 95% confidence intervals. For studies that included multiple post-treatment assessments, the first of these was used. Studies were weighted by the inverse variance of their SMD estimate. The *I* ^2^ statistic was used to quantify the degree of between-study heterogeneity. Sensitivity analyses were conducted to determine the stability of the overall SMD when accounting for various sources of between-study heterogeneity, including study design (RCT versus non-randomised study), sample size (*n* > 30 *v*. *n* ≤ 30), treatment duration (single-session interventions versus other interventions) and treatment type (CBT-based interventions versus other interventions). Forest plots were generated displaying SMDs for each study included in meta-analyses, as well as the overall SMD. Tests for publication bias (e.g. Egger’s test for detecting funnel plot asymmetry^26^) were not conducted due to the low number of studies included in meta-analyses.^27^

## Results

### Results of search and article selection process

**Fig. 1:**
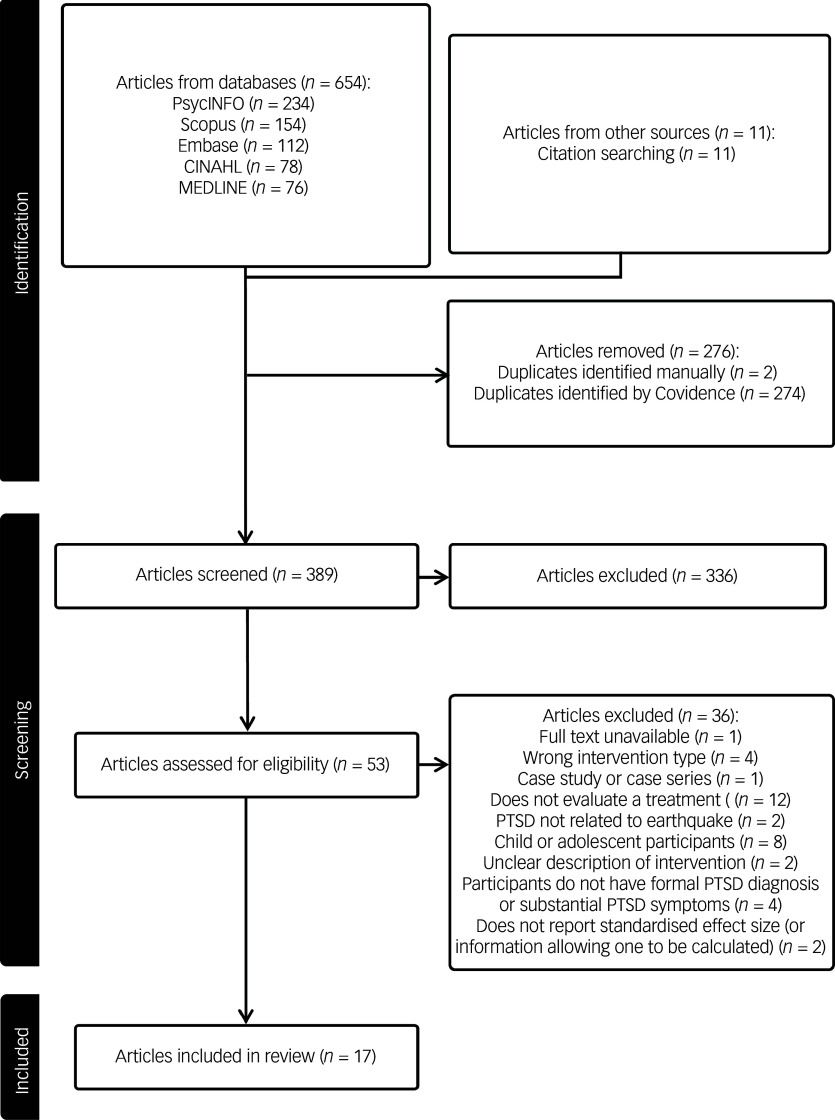
Preferred Reporting Items for Systematic Reviews and Meta-Analyses (PRISMA) flow diagram of the search and article selection process. Articles identified through citation searching are those identified using the reference lists of included studies. PTSD, post-traumatic stress disorder.

Figure 1 displays the PRISMA flow diagram of the search and article selection process. The search identified 665 articles, including 654 identified through database searches and 11 through manual searches of reference lists. After removing 276 duplicates, 389 articles were available for screening. Of these, 339 were excluded at the title and abstract screening stage, leaving 50 articles available for full-text screening. Following that, 17 articles were identified as fulfilling inclusion and exclusion criteria and were included in the narrative synthesis. Of these 17 articles, three^21,28,29^ specified the presence of PTSD symptoms as part of participant inclusion criteria, rather than the presence of a PTSD diagnosis or surpassing an established cut-off on a self-report measure. It was decided to include these studies, because most of their participants were deemed to have substantial PTSD symptoms based on pre-treatment mean scores or rates of diagnosis. Two additional potentially relevant articles were identified,^30,31^ but required further information to determine eligibility; however, the study authors were not contactable. Thus, the review included a total of 17 articles reporting the results of 16 unique studies (2 articles reported on the same RCT; the term ‘article’ henceforth refers to individual publications, while ‘study’ refers to individual trials).

### Study and sample characteristics

Supplementary Table 3 contains the characteristics and key findings of each included study. Among the 17 articles, 9 reported the results of 8 RCTs,^17,18,28,32–37^ 4 used non-randomised designs^19,20,29,38^ and 4 used non-controlled designs.^21,22,39,40^ Six studies were conducted in Turkey, four in China, two in Italy and one in each of Chile, Indonesia, Iran and Mexico. The sample size in each study ranged from 10 to 529 participants, with the review representing a total of 1315 participants receiving a psychological intervention. The proportion of female participants ranged from 30.6 to 100%, and 10 of the 16 studies contained samples in which at least 75% of participants were female. Participants’ mean age (in the 14 articles reporting mean age of the sample) ranged from 25 to 72 years. Two studies focused on specific age groups: Bianchini et al^29^ evaluated CBT for younger adults (aged 16–30 years), and Efendi et al^34^ evaluated this for older adults (aged 60+ years).

### Intervention type and characteristics

Eleven studies evaluated CBT-based interventions. Three studies focused on interventions broadly defined as CBT,^29,34,38^ which utilised various CBT components (e.g. psychoeducation, exposure, cognitive restructuring). Remaining CBT-based interventions were specific CBT variants. Four studies (five articles)^17,21,32,33,39^ evaluated exposure-based behavioural treatments, utilising both earthquake simulator-assisted exposure and self-exposure exercises. Two studies^36,37^ focused on narrative exposure therapy (NET), a manualised treatment containing elements of exposure and cognitive processing therapy. One study evaluated a problem-solving training intervention,^19^ and another a psychoeducation intervention.^20^ Consistent with prior reviews,^9^ both were classed as CBT-based interventions.

Three studies evaluated EMDR,^18,22,40^ in which participants focused on a memory of the traumatic event while experiencing bilateral stimulation, intended to facilitate processing of the trauma memory.^41^

Two studies evaluated other interventions, one of which^35^ evaluated interpersonal psychotherapy (IPT), which aimed to reduce earthquake-related psychological symptoms by examining and addressing interpersonal relationship issues. The other^28^ evaluated a self-guided, internet-based intervention called My Trauma Recovery; the intervention was based on principles of social cognitive theory and aimed to increase coping self-efficacy.

#### Intervention length and format

All interventions were relatively brief, with 12 or fewer sessions. Four studies evaluated single-session interventions.^17,18,21,32^ In three studies^22,39,40^ the number of sessions varied between participants, and treatment concluded when clinical improvement was noted. Among these studies, the average number of sessions ranged from two to five. The remaining seven studies evaluated treatments with a fixed number of sessions, ranging from 4 to 12. Most interventions (13 of the 16 studies) were delivered in an individual format, apart from one problem-solving intervention^20^ and one CBT intervention,^38^ which were both delivered in group format. In one study, 83% of participants received the treatment individually, 15% in a group and 2% received a combination of both.^39^

### Intervention outcome

#### Randomised controlled trials (*k* = 8)

Two RCTs compared the outcome of single-session, exposure-based behavioural treatment with a waitlist control.^17,32^ Başoğlu et al^32^ trained participants to self-administer exposure, which was designed to reduce fear and avoidance through enhancing participants’ sense of control. The intervention was associated with significantly greater reductions in PTSD (SMD = 0.94), traumatic stress (SMD = 0.78) and depression (SMD = 0.55) symptoms at post-treatment, although analyses of individual PTSD symptoms indicated that significant between-group differences were apparent only for behavioural avoidance.^33^ Başoğlu et al^17^ evaluated a similar intervention, including earthquake simulator-assisted exposure in addition to self-administered exposure. Participants in the intervention group reported significantly greater reductions in PTSD symptoms from baseline to 4 weeks (SMD = 0.93) and 8 weeks (SMD = 0.97) post-treatment. Significantly greater reductions were observed for depression symptoms from baseline to 4 weeks post-treatment (SMD = 1.31), but treatment effects became non-significant at 8 weeks post-treatment.

Two RCTs evaluated NET.^36,37^ Zang et al^36^ randomised participants to a NET or waitlist control condition. Those who received NET reported significantly less severe PTSD (SMD ranged from 1.61 for intrusion to 2.67 for hyperarousal), anxiety (SMD = 1.16) and depression (SMD = 1.40) symptoms at post-treatment when controlling for baseline symptoms. Zang et al^37^ compared NET, NET-R (a revised version thought to be more suitable for individuals exposed to a single traumatic event) and a waitlist condition. Both NET and NET-R were associated with less severe PTSD, depression and anxiety symptoms at post-treatment compared with controls (SMD ranged from 1.24 to 7.41 for NET versus control, and from 1.04 to 3.77 for NET-R versus control). No significant differences were observed between NET and NET-R.

Efendi et al^34^ evaluated trauma-focused CBT among 90 older adults. At post-treatment, significantly fewer participants continued to meet criteria for PTSD in the intervention group (17.8%) than in the control group (88.9%). Participants who received CBT also reported significantly less severe depression symptoms at post-treatment (SMD = 1.78).

Jarero et al^18^ evaluated a single session of EMDR in a small sample of 18 participants. Participants who received EMDR reported significantly greater reductions in PTSD symptoms from baseline to post-treatment compared with waitlist participants (SMD = 3.43).

Jiang et al^35^ compared the outcome of IPT and treatment as usual (consisting of medication management and crisis counselling) with treatment as usual only. Participants who received IPT and treatment as usual experienced significantly greater reductions in rates of PTSD diagnoses from baseline to post-treatment (66.7 to 13.6%) compared with those who received treatment as usual only (45.5 to 42.1%).

Wang et al^28^ evaluated a self-guided, internet-based intervention. Participants who received the intervention reported significantly less severe PTSD symptoms at post-treatment compared with control participants (SMD = 0.71), although no significant differences were observed for depression symptoms (SMD = 0.02).

#### Non-randomised studies (*k* = 4)

Two non-randomised studies compared the outcome of CBT-based interventions with that of receiving no intervention. Bianchini et al^29^ reported that participants who received CBT had significantly greater reductions in overall PTSD severity from baseline to post-treatment compared with waitlist controls (SMD = 0.58). Analyses of individual symptom clusters indicated significant between-group differences for avoidance (SMD = 0.32), but neither for hyperarousal (SMD = −0.01) nor re-experiencing (SMD = 0.07) symptoms. Ferdos and Seyed-Hossein^19^ evaluated a problem-solving training programme; participants in the intervention group reported significantly greater reductions in PTSD symptoms from baseline to post-treatment compared with controls (SMD = 1.41).

Both remaining non-randomised studies compared a psychological intervention with another active intervention. Leiva-Bianchi et al^38^ compared the outcome of group CBT with an abbreviated version of the same intervention, delivered over a shorter time frame and excluding homework assignments between sessions. Participants who received complete CBT reported significant reductions in PTSD severity, while no significant improvements were observed among those who received the abbreviated treatment (SMD = 7.12).

Oflaz et al^20^ compared the outcome of three treatments: psychoeducation; psychoeducation and medication; and medication only. All three groups experienced significant reductions in PTSD and depression symptoms from baseline to post-treatment. Participants who received psychoeducation only reported significantly less severe PTSD symptoms at post-treatment than those who received medication only (SMD = 0.69), although no differences were observed between those who received psychoeducation only and those who received both psychoeducation and medication (SMD = −0.20). No significant differences in post-treatment depression symptoms were observed between those who received psychoeducation only and those who received medication only (SMD = −0.03), or both psychoeducation and medication (SMD = −1.09).

#### Non-controlled studies (*k* = 4)

Two non-controlled studies evaluated exposure-based behavioural treatments. Başoğlu et al^21^ evaluated a single session of earthquake simulator-assisted exposure among ten participants. Significantly less severe traumatic stress and depression symptoms were found at 2, 4, 8 and 12 weeks post-treatment relative to baseline. However, treatment effects were not large (i.e. *d* > 0.80) until 4 weeks post-treatment for traumatic stress. Significant reductions in PTSD symptoms were observed from baseline to 12 weeks post-treatment (*d* = 2.50; PTSD symptoms assessed only at 12 weeks post-treatment). In a larger open trial (*n* = 231), Başoğlu et al^39^ evaluated a similar exposure-based treatment that included participants self-adminstering exposure. Participants reported significantly less severe traumatic stress and depression symptoms at post-treatment relative to baseline, with slightly larger effects for those with PTSD (*d* = 1.80 and 1.20 for traumatic stress and depression symptoms, respectively) than for those with subthreshold symptoms (*d* = 1.40 and 0.80, respectively).

Two non-controlled studies evaluated EMDR. Konuk et al^40^ reported significantly less severe PTSD symptoms at post-treatment (*d* = 3.08), with improvements observed for all three symptom clusters. Saltini et al^22^ reported significantly less severe PTSD symptoms at post-treatment (*d* = 1.57 for particiants treated within 1 month post-earthquake; *d* = 1.43 for those treated after 1 month), and significant improvements were observed for all three symptom clusters.

### Quality assessment

Table 1 displays the results of quality assessments for the RCTs. Studies were generally of high methodological quality, although some did not report sufficient detail for a definitive judgement to be made for all domains. In most studies, randomisation was performed appropriately, intervention and control groups were comparable at baseline, complete outcome data were available and participants adhered to their assigned intervention. However, most RCTs (five of the eight studies) did not blind outcome assessors to participants’ assigned experimental condition, largely due to the use of self-report measures for assessment of treatment outcome.

**Table 1:**
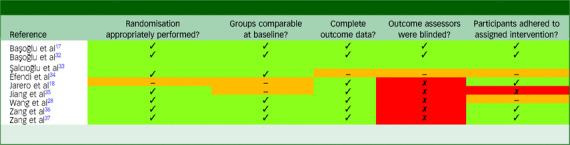
Results of quality assessments for randomised controlled trials

Quality assessments were conducted using the Mixed Methods Appraisal Tool. Green cells indicate ‘Yes’, red cells indicate ‘No’ and orange cells indicate ‘Can’t tell’ (i.e. that insufficient information was reported to make a definitive judgement).

Table 2 displays the results of quality assessments for the non-randomised and non-controlled studies. The quality of these studies was more variable. Among the four non-randomised studies, only one contained a sample that was probably representative of all individuals who developed PTSD following the earthquake. Two studies had complete outcome data, and two did not report sufficient information (complete outcome data were not assumed in the absence of information pertaining to attrition). All studies used appropriate outcome measurements, either clinician-administered diagnostic interviews or validated self-report measures. Confounding (e.g. arising from between-group differences at baseline) was adequately accounted for in two of the four studies. In all non-randomised studies, it was not clear whether participants adhered to the intervention as intended.

**Table 2:**
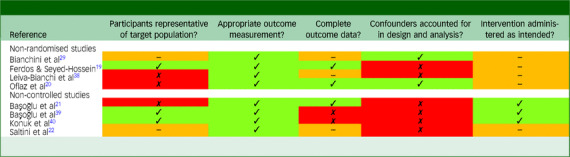
Results of quality assessment for non-randomised and non-controlled studies

Quality assessments were conducted using the Mixed Methods Appraisal Tool. Green cells indicate ‘Yes’, red cells indicate ‘No’ and orange cells indicate ‘Can’t tell’ (i.e. that insufficient information was reported to make a definitive judgement).

Among the four non-controlled studies, two contained samples that were likely to be representative. One study had complete outcome data, two did not have complete data and one did not report sufficient information. All studies used appropriate outcome measures. All non-controlled studies were rated as not accounting for bias arising from confounding, because the primary limitation associated with non-controlled designs is that pre- to post-treatment changes may be attributable to other unmeasured factors rather than to the intervention. The intervention was administered as intended in three of the four non-controlled studies.

### Meta-analytic results

#### PTSD symptoms

**Fig. 2:**
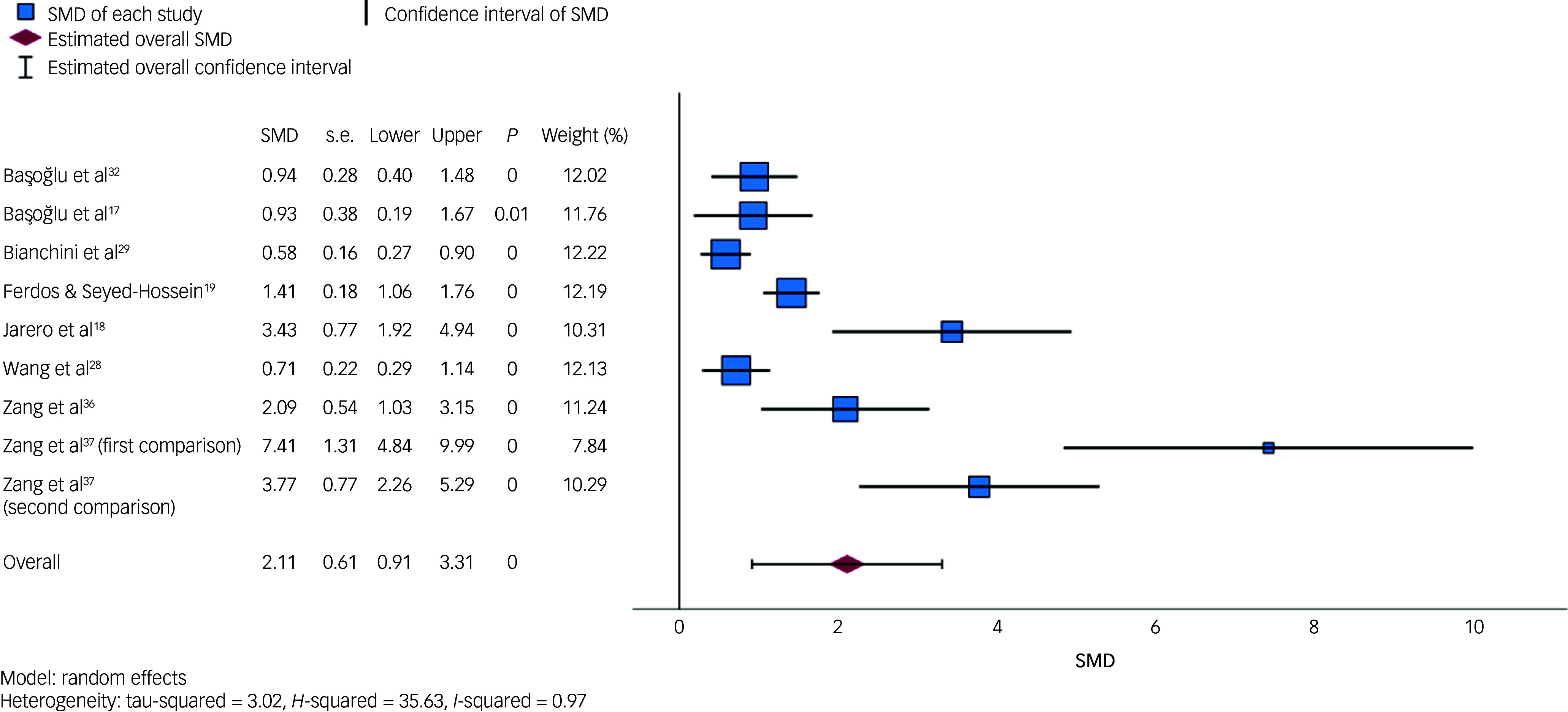
Forest plot displaying individual study standardised mean differences (SMDs) and pooled random effects estimate for post-traumatic stress disorder symptoms. The forest plot displays SMDs, standard errors (s.e.), lower and upper 95% confidence intervals, *P*-values and weights for each included comparison, as well as the pooled estimate. For the first comparison in Zang et al,^37^ the intervention group was narrative exposure therapy; for the second comparison in Zang et al,^37^ the intervention group was revised narrative exposure therapy. Increasing SMD values (>0) favour the intervention.

Eight studies (six RCTs and two non-randomised studies, totalling nine comparisons) compared the outcomes of receiving a psychological intervention versus receiving no intervention (i.e. waitlist; see Supplementary Table 3). Figure 2 is a forest plot displaying the SMDs for each study and the pooled random effects estimate. The overall combined SMD was 2.11 (95% CI = 0.92, 3.31, *P* < 0.001), and was associated with a considerable degree of between-study heterogeneity (*I* ^2^ = 97.19%).

Sensitivity analyses indicated that the pooled estimate was relatively robust to differences in study design (RCT versus non-randomised design), intervention duration (single-session interventions versus other interventions) and treatment type (CBT-based interventions versus other interventions). The overall SMD was 2.53 (95% CI = 0.96, 4.09, *P* = 0.002, *I* ^2^ = 96.84%) when excluding two non-randomised studies; 2.43 (95% CI = 0.60, 4.26, *P* = 0.009, *I* ^2^ = 98.46%) when excluding three studies of single-session interventions; and 2.20 (95% CI = 0.69, 3.72, *P* = 0.004, *I* ^2^ = 97.72%) when excluding two studies of non-CBT-based interventions. However, excluding three studies (four comparisons) containing 30 participants or fewer resulted in a considerably smaller overall estimate of 0.91 (95% CI = 0.58, 1.25, *P* < 0.001) and reduced between-study heterogeneity (*I* ^2^ = 64.65%).

A separate analysis was also conducted excluding the study by Bianchini et al.^29^ That study reported very small standard deviations associated with pre- and post-treatment mean scores, resulting in unfeasibly large effect sizes, and was excluded from prior meta-analyses^16^ because its inclusion skewed the results considerably. We believe the standard deviations reported in this study are likely to be standard error values, and we therefore converted these values to standard deviations prior to meta-analyses (see Supplementary Table 3 footnote for further information). Excluding this study from meta-analyses did not substantially alter the pooled estimate (SMD = 2.34, 95% CI = 1.02, 3.66) or degree of heterogeneity (*I* ^2^ = 96.49%).

#### Depression symptoms

**Fig. 3:**
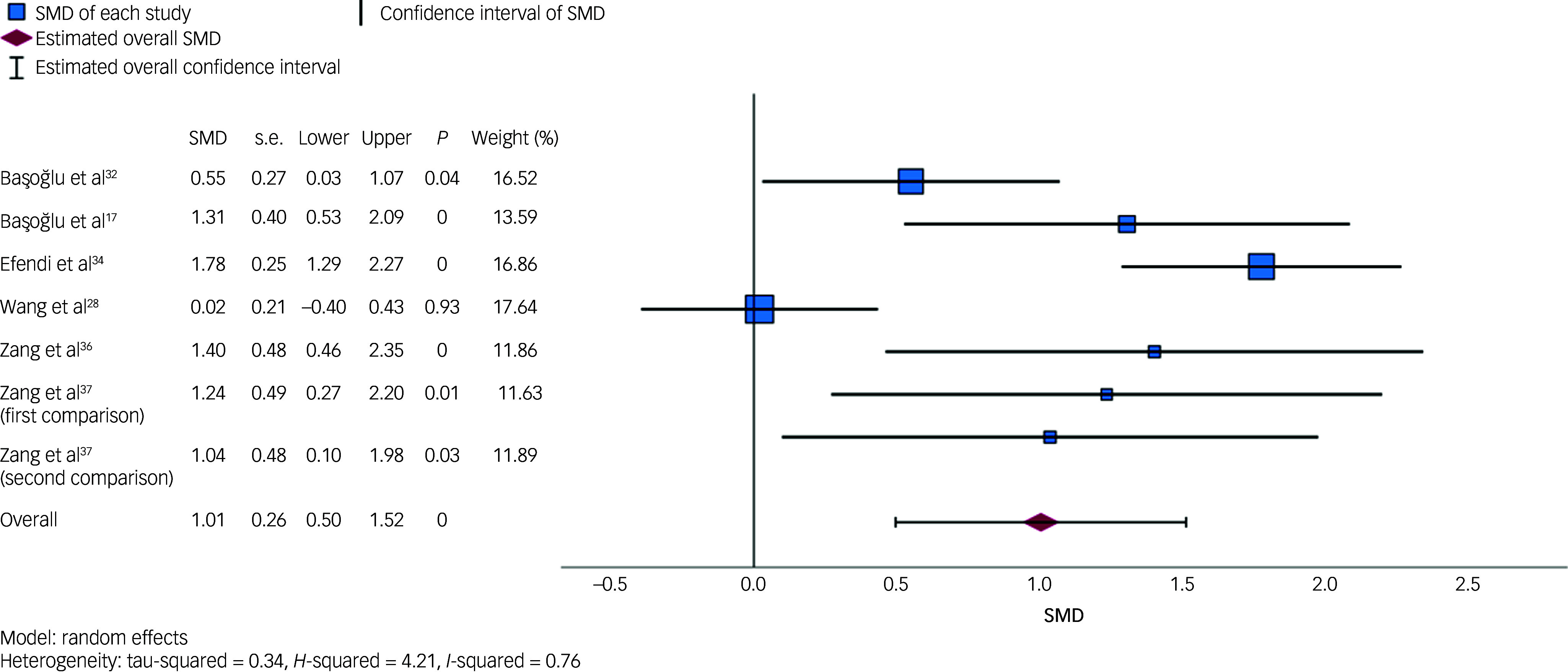
Forest plot displaying individual study standardised mean differences (SMDs) and pooled random effects estimate for depression symptoms. The forest plot displays SMDs, standard errors (s.e.), lower and upper 95% confidence intervals, *P*-values and weights for each included comparison, as well as the pooled estimate. For the first comparison in Zang et al,^37^ the intervention group was narrative exposure therapy; for the second comparison Zang et al,^37^ the intervention group was revised narrative exposure therapy. Increasing SMD values (>0) favour the intervention.

Six RCTs (containing seven comparisons) compared the outcome of receiving a psychological intervention with that of receiving no intervention (i.e. waitlist; see Supplementary Table 3). Figure 3 is a forest plot displaying SMDs for each study and the pooled random effects estimate. The overall SMD was 1.01 (95% CI = 0.50, 1.52, *P* < 0.001), and was associated with a substantial degree of between-study heterogeneity (*I* ^2^ = 76.22%).

Sensitivity analyses indicated that the pooled estimate was relatively robust across differences in sample size (*n* > 30 *v*. *n* ≤ 30) and intervention duration (single-session interventions versus other interventions). The overall SMD was 0.90 (95% CI = 0.11, 1.69, *P* = 0.026, *I* ^2^ = 88.82%) when excluding studies of samples containing 30 participants or fewer; and 1.06 (95% CI = 0.38, 1.74, *P* = 0.002, *I* ^2^ = 80.05%) when excluding two studies of single-session interventions. The overall SMD was slightly larger when excluding one study of a non-CBT-based intervention (SMD = 1.22, 95% CI = 0.78, 1.65, *P* < 0.001), and was associated with less between-study heterogeneity (*I* ^2^ = 53.35%). Because all included comparisons came from RCTs, sensitivity analyses relating to study design were not conducted.

## Discussion

The current review identified 16 studies (reported in 17 articles) evaluating psychological interventions for earthquake-related PTSD in adults, representing 1315 participants across 7 countries. Studies reported statistically significant treatment effects with moderate to large effect sizes. Large overall SMD estimates were found for PTSD and depression symptoms in meta-analyses. However, estimates for both were associated with relatively wide confidence intervals and substantial heterogeneity, and should be interpreted with caution.

Psychological interventions were consistently associated with greater reductions in PTSD severity than waitlist controls. However, only two studies^37,38^ directly compared the outcome of two psychological interventions (CBT versus abbreviated CBT; NET versus NET-R), and no studies directly compared different intervention types (e.g. CBT versus EMDR). Additionally, we were unable to meaningfully compare the outcome of different psychological interventions using meta-analyses, because few studies were eligible and these evaluated a range of interventions. Consistent with prior syntheses,^16^ NET was associated with relatively larger effect sizes although both studies of NET used small samples (*n* ≤ 30) of participants from one country (China), and it is unclear whether these findings generalise to other earthquake-exposed groups. Meta-analyses of trauma-exposed individuals more generally have found either no significant differences^42^ or small differences^43^ between NET and other active control conditions (including other psychological interventions). Thus, additional studies comparing therapies are needed before strong conclusions can be drawn about the relative efficacy of different psychological interventions for earthquake-related PTSD.

Similarly, only two studies compared psychological interventions with non-psychological treatments. These both reported evidence for the superiority of psychological interventions over pharmacological treatment,^20^ and over the combination of medication management and crisis counselling.^35^ However, these were insufficient to draw strong conclusions. Prior meta-analyses have concluded that psychological and pharmacological interventions for PTSD are associated with comparable outcomes at post-treatment, although psychological interventions have been associated with better long-term outcomes.^44^ Additional studies comparing psychological and non-psychological interventions are required to clarify whether these findings generalise to earthquake-exposed individuals.

The overall SMD for PTSD symptoms was associated with considerable heterogeneity (*I* ^2^ = 97.19%), indicating that the variability among study results was much higher than would be expected by chance.^45^ Sensitivity analyses indicated that the *I* ^2^ estimate was not strongly influenced by differences in study design, intervention duration or treatment type, suggesting that other factors (such as disaster-specific contextual factors) contributed to high degrees of heterogeneity. The *I* ^2^ estimate decreased considerably (*I* ^2^ = 64.65%) when excluding the four effect sizes from studies with small samples (*n* ≤ 30), suggesting that between-study differences in sample size contributed to effect size variation. This may also indicate some potential publication bias associated with the smaller studies. It is possible that smaller studies with small or non-significant results may not have been published.

Removing small studies from meta-analyses also resulted in a lower overall SMD for PTSD symptoms (SMD = 0.91), indicating that smaller studies tended to report larger SMDs. Three of the four comparisons from small studies evaluated NET, which was associated with larger effect sizes and may partially account for the reduced overall SMD. Alternatively, small studies may be more prone to publication bias, or may be more likely to be of lower methodological quality, both of which could account for larger potentially biased treatment effects.^46^ However, a recent review of psychotherapies for earthquake-related PTSD found little evidence of publication bias,^16^ and our quality assessment found no clear association between sample size and methodological quality. Small studies may also be characterised by more tightly controlled conditions (e.g. more homogenous samples, increased treatment fidelity), contributing to larger effects, although these study settings may be less generalisable to real-world clinical practice.

Psychological interventions were also effective in reducing depression symptoms, although to a lesser extent, as indicated by the smaller overall SMD estimate of 1.01. The review excluded interventions primarily designed to target depression symptoms, which may have contributed to the smaller overall effect size for depression. Additionally, only one study required participants to report substantial depression symptoms in addition to PTSD symptoms, and several excluded participants with primary depression, which may have attenuated effect sizes. Despite this, most studies that included a measure of depression reported statistically significant treatment effects, excluding one study of an internet-based intervention^28^ and one of a psychoeducation intervention,^20^ in which reductions in the intervention group were not significantly different from those observed among controls. One RCT evaluating an exposure-based behavioural treatment^17^ reported significant treatment effects for depression symptoms at the first post-treatment assessment (4 weeks post-treatment), but not at the second (8 weeks post-treatment).

Only two studies included measures of anxiety, both of which evaluated NET among Chinese participants with mild to moderate anxiety levels.^36,37^ NET was associated with significantly greater reductions in anxiety compared with no treatment, suggesting that this intervention is effective for treating comorbid anxiety symptoms. However, too few studies were identified to conduct meta-analyses, and further studies evaluating different psychological interventions are required to draw firm conclusions.

Few studies have investigated long-term outcomes. Results from non-controlled studies suggested that psychological interventions are associated with sustained improvements up to 6 months post-treatment.^40^ Among the RCTs and non-randomised studies, only one^17^ included more than one post-treatment between-group comparison. Exposure to ongoing disruption (e.g. aftershocks, bereavement, displacement) could compromise the long-term efficacy of psychological interventions, because prior PTSD sensitises individuals to the effects of subsequent stressors.^47^ However, psychological interventions may promote resilience,^48^ allowing for maintenance of treatment gains despite ongoing disruption, consistent with findings from the non-controlled studies. Additional studies with longer follow-up periods, particularly RCTs utilising between-group comparisons, are required to clarify long-term outcomes.

Several studies have acknowledged the need for efficient treatment options that could be scaled to address widespread need, and these evaluated single-session (*k* = 4), group-based (*k* = 2) and self-guided, internet-based (*k* = 1) interventions, all of which were effective. In meta-analyses, excluding studies of single-session interventions did not substantially alter the overall SMDs for PTSD or depression, suggesting that the outcomes of these interventions were comparable to more resource-intensive approaches. Group-based treatments were also associated with positive outcomes, although both studies of group-based interventions utilised active comparison groups, making it difficult to compare their outcome with studies utilising waitlist controls. The study of a self-guided, internet-based intervention was associated with a SMD of 0.71 for PTSD symptoms,^28^ which was substantially lower than the overall SMD derived from meta-analyses. The authors suggested that the relatively small treatment effect may have resulted from low levels of participant motivation, or from some participants’ unfamiliarity with computers and the internet. Possibly, lack of therapist contact also contributed to smaller effects.

Consistent with prior reviews,^9^ there was a focus on exposure techniques among the CBT-based interventions. Six studies either utilised exposure exclusively or had a predominant exposure component. In line with prior meta-analyses,^49,50^ we did not classify EMDR as a CBT-based intervention, although some researchers argue that imaginal exposure is the primary mechanism underlying EMDR^51^ (and exposure is a key component of CBT). It has been suggested that exposure-based interventions, which are conceptually based on fear extinction, could be particularly appropriate for addressing earthquake-related fears and avoidance.^12^ Peritraumatic fear consistently predicts ongoing earthquake-related distress,^52,53^ and earthquake-exposed individuals often report anticipatory fear of recurring aftershocks,^54^ driving behavioural avoidance.^55^ Conversely, others have highlighted the challenges of implementing exposure-based treatments under ongoing threat^13^ (e.g. due to aftershocks), where some degree of avoidance is necessary for maintaining safety. We were not able to meta-analytically compare CBT-based interventions with and without an exposure focus, although this may be possible in future meta-analyses when additional studies are available. No obvious differences emerged in narrative syntheses, consistent with some research reporting comparable outcomes between psychotherapies with and without exposure.^56^

The review findings have implications for post-earthquake mental health responses. Psychological interventions are effective for earthquake-related PTSD, and should be offered to those who develop substantial symptoms. The most evidence currently exists for CBT-based interventions (particularly those with an exposure focus), which we recommend as first-line treatments. Although exposure-based interventions were associated with good outcomes, they may be poorly tolerated by some individuals^57^ who may benefit from CBT without exposure. Other interventions such as EMDR and IPT are also effective, but have been the focus of less research. Compared with CBT, fewer clinicians are likely to have expertise in these modalities, which is likely to pose challenges when treatment need is widespread. We also recommend efficient treatment formats, including single-session and group interventions, particularly when many people require treatment and available resources are limited. In resource-limited settings, single-session or group-based CBT interventions could be offered as first-line interventions, with more resource-intensive approaches reserved for those who do not respond to initial treatment. Although self-guided, internet-based interventions offer promise, there is currently insufficient evidence to recommend them as a sole treatment for earthquake-related PTSD, although they could be used to supplement clinician-administered interventions.

Our findings should be interpreted considering limitations of the available evidence. Only half of the included studies were RCTs (*k* = 8), and the implementation of more studies utilising this design is desirable. Quality assessments indicated that the risk of bias across studies was variable. Although the RCTs were generally of high methodological quality, most did not include blinded outcome assessors, which may have introduced observer bias and resulted in inflated effect sizes.^58^ The quality of the non-randomised and non-controlled studies varied; few studies included ostensibly representative samples, and almost none accounted for confounding. Furthermore, many studies did not report sufficient information for a quality assessment to be made for all domains.

Other limitations relate to the review process. Meta-analyses were conducted using only a subset of the included studies that presented summaries suitable for analyses, and there were too few to conduct meaningful subgroup analyses and comparisons. We are therefore unable to draw conclusions about the relative efficacy of different psychological interventions. The MMAT used for quality assessments was chosen because it allowed for the use of a single tool across studies utilising different designs. Although the MMAT has been validated and used extensively, it is not as fine-grained as other quality assessment tools (e.g. the Cochrane Risk of Bias (RoB) tools for RCTs (RoB 2) and non-randomised studies (ROBINS-I). We chose to limit our analysis of RCTs and non-randomised studies to between-groups effects only. Several RCTs conducted pooled within-group analyses to examine longer-term outcomes, which included controls who received the treatment after a waiting period. We did not extract these results, because doing so might have introduced additional heterogeneity (i.e. arising from some participants receiving treatment immediately and others after a delay), although these results may have provided additional information about long-term outcomes.

In conclusion, the current review and meta-analysis evaluated psychological interventions for adult earthquake-related PTSD. These interventions are generally associated with good treatment outcomes, with sufficient evidence to recommend CBT-based interventions as first-line treatments. Efficient interventions, including single-session and group-based treatments, are recommended for addressing widespread treatment need. Additional high-quality studies, particularly those comparing different interventions, utilising longer follow-up periods and examining effects on comorbid symptoms, are recommended to advance understanding of psychological interventions for earthquake-related PTSD.

## Supporting information

Woods et al. supplementary material Woods et al. supplementary material

## Data Availability

Data supporting the findings of this study are available on request from the corresponding author.

## Supplementary material

The supplementary material can be found online at https://doi.org/10.1192/bjo.2025.784
