# Supplementary material for: Psychological interventions for adult earthquake-related post-traumatic stress disorder: systematic review and meta-analysis
Source: BJPsych Open. 2025 Jun 5;11(4):e119. doi: 10.1192/bjo.2025.784 (PMC12188231; doi:10.1192/bjo.2025.784)
Supplement: Woods et al. supplementary material [file S2056472425007847sup001.docx]

**Supplementary Table 1**

*PRISMA Checklist*

| **Section and Topic** | **Item #** | **Checklist item** | **Location where item is reported** |
| --- | --- | --- | --- |
| **TITLE** | | |  |
| Title | 1 | Identify the report as a systematic review. | pg. 4 |
| **ABSTRACT** | | |  |
| Abstract | 2 | See the PRISMA 2020 for Abstracts checklist. | pg. 3 |
| **INTRODUCTION** | | |  |
| Rationale | 3 | Describe the rationale for the review in the context of existing knowledge. | pp. 4-5 |
| Objectives | 4 | Provide an explicit statement of the objective(s) or question(s) the review addresses. | pg. 5 |
| **METHODS** | | |  |
| Eligibility criteria | 5 | Specify the inclusion and exclusion criteria for the review and how studies were grouped for the syntheses. | pg. 6; pp. 8-9 |
| Information sources | 6 | Specify all databases, registers, websites, organisations, reference lists and other sources searched or consulted to identify studies. Specify the date when each source was last searched or consulted. | pg. 6 |
| Search strategy | 7 | Present the full search strategies for all databases, registers and websites, including any filters and limits used. | Suppl. material |
| Selection process | 8 | Specify the methods used to decide whether a study met the inclusion criteria of the review, including how many reviewers screened each record and each report retrieved, whether they worked independently, and if applicable, details of automation tools used in the process. | pg. 7 |
| Data collection process | 9 | Specify the methods used to collect data from reports, including how many reviewers collected data from each report, whether they worked independently, any processes for obtaining or confirming data from study investigators, and if applicable, details of automation tools used in the process. | pg. 7 |
| Data items | 10a | List and define all outcomes for which data were sought. Specify whether all results that were compatible with each outcome domain in each study were sought (e.g. for all measures, time points, analyses), and if not, the methods used to decide which results to collect. | pg. 7 |
|  | 10b | List and define all other variables for which data were sought (e.g. participant and intervention characteristics, funding sources). Describe any assumptions made about any missing or unclear information. | pg. 7 |
| Study risk of bias assessment | 11 | Specify the methods used to assess risk of bias in the included studies, including details of the tool(s) used, how many reviewers assessed each study and whether they worked independently, and if applicable, details of automation tools used in the process. | pg. 8 |
| Effect measures | 12 | Specify for each outcome the effect measure(s) (e.g. risk ratio, mean difference) used in the synthesis or presentation of results. | pg. 8 |
| Synthesis methods | 13a | Describe the processes used to decide which studies were eligible for each synthesis (e.g. tabulating the study intervention characteristics and comparing against the planned groups for each synthesis (item #5)). | pp. 8-9 |
|  | 13b | Describe any methods required to prepare the data for presentation or synthesis, such as handling of missing summary statistics, or data conversions. | pg. 8 |
|  | 13c | Describe any methods used to tabulate or visually display results of individual studies and syntheses. | pg. 9 |
|  | 13d | Describe any methods used to synthesize results and provide a rationale for the choice(s). If meta-analysis was performed, describe the model(s), method(s) to identify the presence and extent of statistical heterogeneity, and software package(s) used. | pg. 8-9 |
|  | 13e | Describe any methods used to explore possible causes of heterogeneity among study results (e.g. subgroup analysis, meta-regression). | pg. 9 |
|  | 13f | Describe any sensitivity analyses conducted to assess robustness of the synthesized results. | pg. 9 |
| Reporting bias assessment | 14 | Describe any methods used to assess risk of bias due to missing results in a synthesis (arising from reporting biases). | N/A (pg. 9) |
| Certainty assessment | 15 | Describe any methods used to assess certainty (or confidence) in the body of evidence for an outcome. | N/A |
| **RESULTS** | | |  |
| Study selection | 16a | Describe the results of the search and selection process, from the number of records identified in the search to the number of studies included in the review, ideally using a flow diagram. | pg. 10; Figure 1 |
|  | 16b | Cite studies that might appear to meet the inclusion criteria, but which were excluded, and explain why they were excluded. | pg. 10 |
| Study characteristics | 17 | Cite each included study and present its characteristics. | pg. 10; Table 1 |
| Risk of bias in studies | 18 | Present assessments of risk of bias for each included study. | pp. 15-16 |
| Results of individual studies | 19 | For all outcomes, present, for each study: (a) summary statistics for each group (where appropriate) and (b) an effect estimate and its precision (e.g. confidence/credible interval), ideally using structured tables or plots. | Suppl. Table 3 |
| Results of syntheses | 20a | For each synthesis, briefly summarise the characteristics and risk of bias among contributing studies. | Tables 1 and 2 |
|  | 20b | Present results of all statistical syntheses conducted. If meta-analysis was done, present for each the summary estimate and its precision (e.g. confidence/credible interval) and measures of statistical heterogeneity. If comparing groups, describe the direction of the effect. | pp. 16-17 |
|  | 20c | Present results of all investigations of possible causes of heterogeneity among study results. | pp. 16-17 |
|  | 20d | Present results of all sensitivity analyses conducted to assess the robustness of the synthesized results. | pp. 16-17 |
| Reporting biases | 21 | Present assessments of risk of bias due to missing results (arising from reporting biases) for each synthesis assessed. | N/A |
| Certainty of evidence | 22 | Present assessments of certainty (or confidence) in the body of evidence for each outcome assessed. | N/A |
| **DISCUSSION** | | |  |
| Discussion | 23a | Provide a general interpretation of the results in the context of other evidence. | pp. 17-23 |
|  | 23b | Discuss any limitations of the evidence included in the review. | pp. 22-23 |
|  | 23c | Discuss any limitations of the review processes used. | pp. 22-23 |
|  | 23d | Discuss implications of the results for practice, policy, and future research. | pp. 21-22 |
| **OTHER INFORMATION** | | |  |
| Registration and protocol | 24a | Provide registration information for the review, including register name and registration number, or state that the review was not registered. | pg. 5 |
|  | 24b | Indicate where the review protocol can be accessed, or state that a protocol was not prepared. | pg. 5 |
|  | 24c | Describe and explain any amendments to information provided at registration or in the protocol. | pp. 6 |
| Support | 25 | Describe sources of financial or non-financial support for the review, and the role of the funders or sponsors in the review. | pg. 1 |
| Competing interests | 26 | Declare any competing interests of review authors. | pg. 2 |
| Availability of data, code and other materials | 27 | Report which of the following are publicly available and where they can be found: template data collection forms; data extracted from included studies; data used for all analyses; analytic code; any other materials used in the review. | pg. 1 |

**Supplementary Table 2**

*Full Database Search Strategy*

| **Database** | **Search Strategy** | |
| --- | --- | --- |
| **PsycINFO** | S1 | earthquake* |
|  | S2 | DE "Posttraumatic Stress Disorder" OR DE "Complex PTSD" |
|  | S3 | posttraumatic stress OR PTSD OR post-traumatic stress OR traumatic stress |
|  | S4 | S2 OR S3 |
|  | S5 | S1 AND S4 |
|  | S6 | DE "Behavior Therapy" OR OR DE "Behavior Modification" OR DE "Cognitive Behavior Therapy" OR DE "Acceptance and Commitment Therapy" OR DE "Cognitive Analytic Therapy" OR DE "Cognitive Processing Therapy" OR DE "Cognitive Techniques" OR DE "Cognitive Therapy" OR DE "Mindfulness-Based Cognitive Therapy" OR DE "Trauma-Focused Cognitive Behavior Therapy" OR DE "Dialectical Behavior Therapy" OR DE "Exposure Therapy" OR DE "Exposure and Response Prevention Therapy" OR DE "Imaginal Exposure" OR DE "In Vivo Exposure" OR DE "Prolonged Exposure Therapy" OR DE "Systematic Desensitization Therapy" OR DE "Virtual Reality Exposure Therapy" OR DE "Eclectic Psychotherapy" |
|  | S7 | DE "Psychotherapy" OR DE "Brief Psychotherapy" OR DE "Emotion Focused Therapy" OR DE "Eye Movement Desensitization Therapy" OR DE "Group Psychotherapy" OR DE "Individual Psychotherapy" OR DE "Interpersonal Psychotherapy" OR DE "Narrative Therapy" OR DE "Psychodynamic Psychotherapy" |
|  | S8 | (intervention OR therap* OR treatment* OR effective*) N1 (psychological OR cognitive OR behavio*) |
|  | S9 | CBT OR CPT OR CT |
|  | S10 | "behavior therapy" OR "behaviour therapy" OR "cognitive therapy" OR "psychotherapy" OR "cognitive processing therapy" OR "exposure therapy" OR "prolonged exposure" OR "eye movement desensiti*" OR "emdr" OR "emotion-focused therapy" OR "emotion focused therapy" OR "imaginal exposure" OR "acceptance and commitment therapy" |
|  | S11 | S6 OR S7 OR S8 OR S9 OR S10 |
|  | S12 | S5 AND S1 |
|  | | |
| **MEDLINE** | S1 | Earthquakes/ |
|  | S2 | earthquake*.ti,ab,kf. |
|  | S3 | S1 or S2 |
|  | S4 | stress disorders, post-traumatic/ or stress disorders, traumatic, acute/ |
|  | S5 | (posttraumatic stress or PTSD or post-traumatic stress or traumatic stress).ti,ab,kf. |
|  | S6 | S4 or S5 |
|  | S7 | behavior therapy/ or cognitive behavioral therapy/ or cognitive restructuring/ or cognitive remediation/ or desensitization, psychologic/ or eye movement desensitization reprocessing/ or virtual reality exposure therapy/ or narrative therapy/ or psychotherapy, brief/ or psychotherapy, psychodynamic/ or emotion-focused therapy/ or psychotherapy, group/ or dialectical behavior therapy/ |
|  | S8 | ("behavior therapy" or "behaviour therapy" or "cognitive therapy" or "psychotherapy" or "cognitive processing therapy" or "exposure therapy" or "prolonged exposure" or "eye movement desensiti*" or "emdr" or "emotion-focused therapy" or "emotion focused therapy" or "imaginal exposure" or "acceptance and commitment therapy").ti,ab,kf. |
|  | S9 | psychotherapy/ or narrative therapy/ |
|  | S10 | ((intervention or therap* or treatment* or effective*) adj1 (psychological or cognitive or behavio*)).ti,ab,kf. |
|  | S11 | (CBT or CPT or CT).ti,ab,kf. |
|  | S12 | S7 or S8 or S9 or S10 or S11 |
|  | S13 | S3 and S6 and S12 |
|  | | |
| **EMBASE** | S1 | earthquake/ |
|  | S2 | earthquake*.ti,ab. |
|  | S3 | S1 or S2 |
|  | S4 | posttraumatic stress disorder/ |
|  | S5 | (posttraumatic stress or PTSD or post-traumatic stress or traumatic stress).ti,ab. |
|  | S6 | S4 or S5 |
|  | S7 | cognitive behavioral therapy/ or behavior therapy/ or cognitive therapy/ or cognitive processing therapy/ or cognitive restructuring/ or mindfulness-based cognitive therapy/ or trauma-focused cognitive behavioral therapy/ |
|  | S8 | exposure therapy/ or behavior therapy/ or "desensitization (psychology)"/ or imaginal exposure therapy/ or systematic desensitization/ or virtual reality exposure therapy/ |
|  | S9 | psychotherapy/ or "eye movement desensitization and reprocessing"/ or group therapy/ or interpersonal psychotherapy/ or narrative therapy/ or psychodynamic psychotherapy/ or emotion-focused therapy/ or short term psychotherapy/ or dialectical behavior therapy/ |
|  | S10 | (CBT or CPT or CT).ti,ab. |
|  | S11 | ("behavior therapy" or "behaviour therapy" or "cognitive therapy" or "psychotherapy" or "cognitive processing therapy" or "exposure therapy" or "prolonged exposure" or "eye movement desensiti*" or "emdr" or "emotion-focused therapy" or "emotion focused therapy" or "imaginal exposure" or "acceptance and commitment therapy").ti,ab. |
|  | S12 | ((intervention or therap* or treatment* or effective*) adj1 (psychological or cognitive or behavio*)).ti,ab. |
|  | S13 | S7 or S8 or S9 or S10 or S11 or S12 |
|  | S14 | S3 and S6 and S13 |
|  | | |
| **CINAHL** | S1 | earthquake* |
|  | S2 | (MH "Stress Disorders, Post-Traumatic") |
|  | S3 | posttraumatic stress OR PTSD OR post-traumatic stress OR traumatic stress |
|  | S4 | S2 OR S3 |
|  | S5 | (MH "Cognitive Therapy") OR (MH "Cognitive Restructuring") OR (MH "Behavior Therapy") |
|  | S6 | (MH "Cognitive Therapy") OR (MH "Cognitive Restructuring") OR (MH "Behavior Therapy") |
|  | S7 | (MH "Desensitization, Psychologic") |
|  | S8 | (MH "Acceptance and Commitment Therapy") OR (MH "Cognitive Restructuring") |
|  | S9 | (MH "Virtual Reality Exposure Therapy") |
|  | S10 | (MH "Psychotherapy") OR (MH "Behavior Modification") OR (MH "Interpersonal Psychotherapy") OR (MH "Psychotherapy, Brief") OR (MH "Psychotherapy, Psychodynamic") OR (MH "Dialectical Behavior Therapy") |
|  | S11 | (MH "Eye Movement Desensitization and Reprogramming") |
|  | S12 | (MH "Psychotherapy, Group") |
|  | S13 | (intervention OR therap* OR treatment* OR effective*) N1 (psychological OR cognitive OR behavio*) |
|  | S14 | CBT OR CPT OR CT |
|  | S15 | "behavior therapy" OR "behaviour therapy" OR "cognitive therapy" OR "psychotherapy" OR "cognitive processing therapy" OR "exposure therapy" OR "prolonged exposure" OR "eye movement desensiti*" OR "emdr" OR "emotion-focused therapy" OR "emotion focused therapy" OR "imaginal exposure" OR "acceptance and commitment therapy" |
|  | S16 | S5 OR S6 OR S7 OR S8 OR S9 OR S10 OR S11 OR S12 OR S13 OR S14 OR S15 |
|  | S17 | S1 AND S4 AND S16 |
|  | | |
| **Scopus** | (TITLE-ABS-KEY(earthquake*)) AND (TITLE-ABS-KEY("posttraumatic stress" OR PTSD OR "post-traumatic stress" OR "post-traumatic stress" OR "traumatic stress")) AND ((TITLE-ABS-KEY((intervention OR therap* OR treatment* OR effective*) W/1 (psychological OR cognitive OR behavio*))) OR (TITLE-ABS-KEY(CBT OR CPT OR CT)) OR (TITLE-ABS-KEY("behavior therapy" OR "behaviour therapy" OR "cognitive therapy" OR "psychotherapy" OR "cognitive processing therapy" OR "exposure therapy" OR "prolonged exposure" OR "eye movement desensiti*" OR EMDR OR "emotion-focused therapy" OR "emotion focused therapy" OR "imaginal exposure" OR "acceptance and commitment therapy"))) | |

**Supplementary Table 3**

*Characteristics and Key Findings of RCTs, Non-Randomised, and Non-Controlled Studies*

| **Author (date)** | **Country** | ***n*** | **Participants** | **Intervention** | **Control** | **Outcomes** | **Assessments** | **Key findings** |
| --- | --- | --- | --- | --- | --- | --- | --- | --- |
| **Randomised controlled trials** | | | | | | | | |
| Başoğlu et al. (2005); Şalcioğlu et al. (2007) | Turkey | *n* = 59 | Adults with DSM-IV PTSD diagnosis. Mean age was 36.3 years (*SD* = 11.5) and 84.7% were female. | Single-session exposure-based behavioural treatment. Participants trained to self-administer exposure. Sessions were one hour long. | Waitlist | PTSD symptoms (CAPS)  Traumatic stress symptoms (TSSC)  Depression symptoms (BDI) | Baseline; six-weeks post-treatment. | Participants who received behavioural treatment reported significantly greater reductions in PTSD, traumatic stress, and depression symptoms from baseline to six-weeks post-treatment (SMD = 0.94 for PTSD; SMD = 0.78 for traumatic stress; SMD = 0.55 for depression). Further analyses of individual PTSD symptoms indicated significant between-group differences were only apparent for behavioural avoidance at six-weeks post-treatment. |
| Başoğlu et al. (2007) | Turkey | *n* = 31 | Adults with DSM-IV PTSD diagnosis. Mean age was 34 years (*SD* = 11) and 87% were female. | Single-session exposure-based behavioural treatment, using earthquake simulator-assisted exposure and training participants to self-administer exposure. Session continued until participants felt in control of their distress (*M*_Duration_ = 33 minutes, *SD* = 18 minutes). | Waitlist | PTSD symptoms (CAPS)  Depression symptoms (BDI) | Baseline; four-weeks post-treatment; eight-weeks post-treatment. | Participants who received behavioural treatment reported significantly greater reductions in PTSD symptoms at four- and eight-weeks post-treatment, relative to baseline (SMD = 0.93 at four-weeks post-treatment; SMD = 0.97 at eight-weeks post-treatment). Participants who received behavioural treatment reported significantly greater reductions in depression symptoms from baseline to four-weeks post-treatment (SMD = 1.31), but the treatment effect was non-significant at eight-weeks. |
| Efendi et al. (2020) | Indonesia | *n* = 90 | Older adults with DSM-5 PTSD diagnosis. Mean age was 70.8 (*SD* = 8.7) and 71.8 (*SD* = 8.5) in the intervention and control groups. 74% of all participants were female. | TF-CBT. Included psychoeducation, relaxation strategies, social problem solving, cognitive restructuring, exposure, and development of the trauma narrative. 12 sessions, each lasting an hour, delivered over six weeks. | Waitlist | PTSD diagnosis (CAPS-5)  Depression symptoms (GDS) | Baseline; post-treatment. | At post-treatment, there were significantly fewer participants who continued to meet criteria for PTSD in the intervention group (17.8%) than in the control group (88.9%). Participants who received TF-CBT reported significantly less severe depression symptoms at post-treatment (SMD = 1.78). |
| Jarero et al. (2011) | Mexico | *n* = 18 | Adults scoring ≥44 on the IES. Participants’ mean age not reported. 44.4% of participants were female. | Single-session of EMDR-PRECI. Sessions lasted between 80 and 130 minutes. | Waitlist | PTSD symptoms (IES) | Baseline; four-days post-treatment. | Participants who received EMDR experienced significantly greater reductions in PTSD symptoms from baseline to post-treatment (SMD = 3.43). |
| Jiang et al. (2014) | China | *n* = 28^a^ | Adults with DSM-IV PTSD diagnosis. | IPT and TAU. IPT focused on interpersonal disputes, role transitions, and grief/loss. TAU consisted of medication management and crisis counselling.  12 sessions, each lasting an hour, delivered over 12 weeks. | TAU only | PTSD diagnosis (CAPS) | Baseline; post-treatment. | From baseline to post-treatment, there was a significantly greater reduction in the proportion of participants with PTSD among those who received IPT + TAU. Rates of PTSD decreased from 66.7% to 13.6% among participants who received IPT + TAU, and from 45.5% to 42.1% among those who received TAU only. |
| Wang et al. (2013) | China | *n* = 94 | Adults reporting at least two PTSD symptoms on the TSQ/Short Screening Scale for DSM-IV PTSD. Participants’ mean age not reported. 82% of participants were female. | Self-guided internet-based intervention based on social cognitive theory. Included modules on social support, self-talk, relaxation, trauma triggers, unhelpful coping, and seeking professional help.  Participants used the website for at least half an hour, on five separate occasions. | Waitlist | PTSD symptoms (PDS)  Depression symptoms (SCL-D) | Baseline; post-treatment. | At post-treatment, participants who received the internet-based intervention reported significantly less severe PTSD symptoms compared to controls (SMD = 0.71). No significant between-group differences were observed for depression symptoms (SMD = 0.02). |
| Zang et al. (2013) | China | *n* = 22 | Adults with DSM-IV PTSD diagnosis. Mean age was 56.6 (*SD* = 12.2) 54.8 (*SD* = 11.6) in the intervention and control groups. 77.3% of all participants were female. | NET. Patients constructed detailed chronological autobiographies, with focus placed on traumatic experiences. Patients were encouraged to reexperience emotions while reporting events.  Four sessions, each lasting between 60 and 90 minutes, delivered over two weeks. | Waitlist | PTSD symptoms (IES-R)  Anxiety symptoms (HADS)  Depression symptoms (HADS) | Baseline; post-treatment. | Participants who received NET reported significantly less severe avoidance (SMD = 1.75), intrusion (SMD = 1.61), hyperarousal (SMD = 2.67), anxiety (SMD = 1.16), and depression (SMD = 1.40) symptoms at post-treatment (while adjusting for baseline symptoms) than controls. |
| Zang et al. (2014) | China | *n* = 30 | Adults with DSM-IV PTSD diagnosis. Mean age was 53.5 (*SD* = 1.2), 56.5 (*SD* = 1.5), and 50.9 (*SD* = 1.23) in the NET, NET-R, and control groups, respectively. 90% of participants were female. | NET (see Zang et al., 2013); NET-R (similar to NET, but with shorter duration, focus placed on the earthquake narration, and participants did not sign off their written biography). | Waitlist | PTSD symptoms (IES-R)  Anxiety symptoms (HADS)  Depression symptoms (HADS) | Baseline; post-treatment. | Participants who received NET and NET-R reported significantly less severe PTSD (SMD = 7.41 for NET vs. waitlist; SMD = 3.77 for NET-R vs. waitlist), anxiety (SMD = 2.60 for NET vs. waitlist; SMD = 2.08 for NET-R vs. waitlist), and depression (SMD = 1.24 for NET vs. waitlist; SMD = 1.04 for NET-R vs. waitlist) symptoms at post-treatment (adjusting for baseline scores) than controls. The NET and NET-R groups did not differ significantly from each other. |
| **Non-randomised studies** | | | | | | | | |
| Bianchini et al. (2013) | Italy | *n* = 163 | Young adults) reporting at least one symptom of DSM-IV PTSD and a high level of perceived distress. Mean age was 26.9 (*SD* = 1.6) and 14.3 (*SD* = 3.5) in the intervention and control groups. 66.7% of all participants were female. | CBT. Included psychoeducation, anxiety management, cognitive restructuring, and elaboration of trauma memories. 12 sessions delivered once per week. | Waitlist | PTSD symptoms (IES-R) | Baseline; post-treatment. | Participants who received CBT experienced significantly greater reductions in overall PTSD symptoms from baseline to post-treatment (SMD = 0.58)^c^. Analyses of individual symptom clusters indicated statistically significant between-group differences were only observed for avoidance (SMD = 0.32; SMD = −0.01 for hyperarousal; SMD = 0.07 for re-experiencing). |
| Ferdos & Seyed-Hossein (2007) | Iran | *n* = 160 | Adults with DSM-IV PTSD diagnosis. Participants’ mean age not reported. 30.6% of participants were female. | Problem-solving training program delivered in a group format. 12 sessions, each lasting two hours, delivered three times per week for one month. | No intervention | PTSD symptoms (M-PTSD) | Baseline; post-treatment. | Participants who received the problem-solving training program experienced significantly greater reductions in PTSD symptoms from baseline to post-treatment, compared to controls (SMD = 1.41). |
| Leiva-Bianchi et al.  (2018) | Chile | *n* = 13^b^ | Adults reporting at least three severe PTSD symptoms on the SPRINT-E. Mean age was 46 and 51 in the complete and abbreviated CBT groups, respectively. 76.9% of all participants were female. | CBT-PD delivered in a group format. Included psychoeducation, breathing retraining, behavioural activation, and cognitive restructuring. Participants received 10 to 12 sessions, each lasting 60 to 90 minutes, delivered once per week. | Abbreviated CBT-PD^d^ | PTSD symptoms (SPRINT-E) | Baseline; post-treatment. | Participants who received the full CBT intervention reported significant reductions in PTSD symptoms, while those who received the abbreviated treatment did not experience significant improvements (SMD = 7.12). |
| Oflaz et al. (2008) | Turkey | *n* = 51 | Adults with a PTSD diagnosis. Mean age was 32.7 years (*SD* = 12.7) and 62.7% of participants were female. | Psychoeducation intervention. Six sessions, each lasting 60 to 90 minutes, delivered once per week. | Psychoeducation and medication.  Medication only. | PTSD symptoms (CAPS)  Depression symptoms (HDS) | Baseline; post-treatment. | All groups experienced significant reductions in PTSD and depression symptoms from baseline to post-treatment.  Participants who received psychoeducation only reported significantly less severe PTSD symptoms at post-treatment compared to those who received medication only when adjusting for baseline PTSD severity (SMD = 0.69). No significant differences were observed between participants who received psychoeducation only and those who received psychoeducation + medication (SMD = −0.20).  No significant differences in post-treatment depression scores were observed between the psychoeducation only group and the medication only group (SMD = −0.03), or between the psychoeducation group and the psychoeducation + medication group (SMD = −1.09) when adjusting for baseline depression severity |
| **Non-controlled studies** | | | | | | | | |
| Başoğlu et al. (2003a) | Turkey | *n* = 10 | Adults with earthquake-related PTSD symptoms. Mean age was 36 years (*SD* = 8) and all participants were female. | Single-session of exposure-based behavioural treatment using an earthquake simulator. Sessions were one hour long. |  | PTSD symptoms (CAPS; at baseline and 12-weeks post-treatment only)  Traumatic stress symptoms (TSSC)  Depression symptoms (BDI) | Baseline; two-, four-, eight-, and 12-weeks post-treatment. | Participants reported significantly less severe traumatic stress symptoms at all post-treatment assessments, relative to baseline (*d* = 0.80, 2.60, 1.70, and 2.50 at two-, four-, eight-, and 12-weeks post-treatment, respectively).  Participants reported significantly less severe PTSD symptoms at 12-weeks post-treatment, relative to baseline (*d* = 2.50).  Participants reported significantly less severe depression symptoms at all post-treatment assessments, relative to baseline (*d* = 1.20, 1.90, 0.90, and 1.20 at two-, four-, eight-, and 12-weeks post-treatment, respectively). |
| Başoğlu et al. (2003b)^e^ | Turkey | *n* = 231 | Adults with earthquake-related PTSD symptoms, including those with a PTSD diagnosis and those with subthreshold symptoms. Mean age was 35 years (*SD* = 11) and 87% of all participants were female. | Brief exposure-based behavioural treatment. Participants trained to self-administer exposure. Length of intervention was not pre-determined. Treatment concluded when therapist and participant noted improvement. Mean number of sessions was 4.3 (*SD* = 2.6, range = 1 to 17). | - | Traumatic stress symptoms (TSSC)  Depression symptoms (BDI) | All participants completed baseline and at least one post-treatment assessment. Some participants completed follow-up assessments (completed on average 66 days post-treatment). | Participants reported significantly less severe traumatic stress symptoms at their last assessment (*d* = 1.80 among participants with PTSD; *d* = 1.40 among those with subthreshold symptoms) and at follow-up (*d* = 2.30 among those with PTSD; *d* = 1.80 among those with subthreshold symptoms) relative to baseline.  Participants reported significantly less severe depression symptoms at their last assessment (*d* = 1.20 among participants with PTSD; *d* = 0.80 among those with subthreshold symptoms) and at follow-up (*d* = 4.50 among those with PTSD; *d* = 0.90 among those with subthreshold symptoms) relative to baseline |
| Konuk et al. (2006) | Turkey | *n* = 41 | Adults with PTSD diagnosis. Mean age was 43.3 among female participants and 41.2 among male participants. 75.6% of all participants were female. | EMDR.  Number of sessions was variable. Sessions were 90 minutes long, and participants received treatment until improvements were noted. Mean number of sessions was 5 (range = 2 to 12). | - | PTSD symptoms (PSS-SR) | Baseline; post-treatment; six-months post-treatment. | Participants experienced significant reductions in overall PTSD symptoms from pre- to post-treatment (*d* = 3.08) and from pre- treatment to follow-up (*d* = 2.45).  Significant improvements were observed for all symptom clusters from pre-treatment to post-treatment (*d* = 2.57, 2.48, and 2.90 for re-experiencing, avoidance, and hyperarousal symptoms, respectively), and from pre-treatment to follow-up (*d* = 2.23, 1.96, and 1.95 for re-experiencing, avoidance, and hyperarousal, respectively). |
| Saltini et al. (2018) | Italy | *n* = 529 | Adults scoring ≥ 33 on the IES-R and assessed as having DSM-IV PTSD symptoms by a clinician. Mean age was 46.4 years (*SD* = 12.9) and 81.8% of participants were female. | EMDR based on the Recent Traumatic Episode Protocol.  Number of sessions was variable (range = 2 to 4 sessions). | - | PTSD symptoms (IES-R) | Baseline; post-treatment. | Participants reported significant reductions in PTSD severity from baseline to post-treatment (*d* = 1.57 for participants treated within one-month post-earthquake; *d* = 1.43 among participants treated later).  Significant improvements were observed for all symptom clusters (*d* = 0.62 and 0.76 for avoidance; *d* = 1.58 and 1.41 for intrusion; *d* = 1.63 and 1.49 for hyperarousal). |

*Note.* BDI = Beck Depression Inventory, CAPS = Clinician Administered PTSD Scale, CBT = cognitive behavioural therapy, CBT-PD = cognitive behavioural therapy for post-disaster distress, EMDR-PRECI = Eye movement desensitisation and reprocessing Protocol for Recent Critical Incidents, GDS = Geriatric Depression Scale, HADS = Hospital Anxiety and Depression Scale, HDS = Hamilton Depression Scale, IES = Impact of Events Scale, IES-R = Impact of Events Scale-Revised, IPT = interpersonal therapy, M-PTSD = Mississippi PTSD Scale, NET = narrative exposure therapy, NET-R = revised narrative exposure therapy, PDS = Post-Traumatic Diagnostic Scale, PSS-SR = PTSD Symptom Scale Self-Report, SCL-D = Symptom Checklist depression subscale, TAU = treatment as usual, TF-CBT = trauma-focused cognitive behavioural therapy, TSSC = Traumatic Stress Symptom Checklist, TSQ = Trauma Screening Questionnaire.

^a^ Study contained 49 participants overall, but only 28 with PTSD. ^b^ Study contained 29 participants overall, but only 13 with PTSD. ^c^ This study reported extremely small *SD* values, which we believe are likely to be standard errors. Study authors were not contactable to clarify this issue. Thus, these values were first converted to standard deviations (*SD* = *SE ×* √*n*) before calculating effect sizes. ^d^ In the abbreviated treatment, participants did not complete homework tasks and the treatment was condensed into four sessions (each lasting two and a half hours), delivered once per week. ^e^ This study used the TSSC as their primary outcome measure. The TSSC includes items pertaining to PTSD and depression symptoms and is thus not a ‘pure’ measure of PTSD. We chose to include this study because the majority of the TSSC items relate to PTSD symptoms (17 of the total 23 items), and because this study was deemed to be of direct relevance to the research questions addressed in the review.
